# Psychosocial factors that mediate the association between mode of birth and maternal postnatal adjustment: findings from a population-based survey

**DOI:** 10.1186/s12905-019-0738-x

**Published:** 2019-03-04

**Authors:** Fiona Alderdice, Jane Henderson, Charles Opondo, Marci Lobel, Maria Quigley, Maggie Redshaw

**Affiliations:** 1 Policy Research Unit in Maternal Health and Care, National Perinatal Epidemiology Unit, Nuffield Department of Population Health, University of Oxford, Old Road Campus, Headington, Oxford, OX3 7LF UK; 2 Professor of Psychology, Stony Brook University, Stony Brook, NY 11794-2500 USA

**Keywords:** Mode of birth, Maternal postnatal adjustment, Psychosocial, Mediation

## Abstract

**Background:**

Mode of birth has been found to be associated with maternal postnatal adjustment with women who have Caesarean Sections (CS) thought to be at higher risk of emotional distress. However the relationship is complex and studies have demonstrated mixed findings. The aim of this study is to evaluate a model that explores the direct relationship between mode of birth and postnatal maternal adjustment at 3 months and indirect relationships through psychosocial variables.

**Methods:**

A secondary analysis of a population-based survey conducted in England, UK in 2014. The analysis included primiparous women with singleton babies who provided information about mode of birth (*n* = 2139).

**Results:**

Maternal postnatal adjustment, as measured by Maternal postnatal wellbeing and Satisfaction with care during labour and birth, varied by mode of birth. Women who had an unplanned CS had the poorest postnatal adjustment. Mode of birth was not associated with Maternal/infant sense of belonging. Four out of the five proposed mediation variables (Perceived control, Maternal expectation, Support in labour, How long until the mother held her baby), showed partial mediation of the relationship between mode of birth and both Maternal postnatal wellbeing and Satisfaction with care during labour and birth. The strongest mediator was Perceived control and the only variable not to show a significant mediation effect was Health of the infant at 3 months.

**Conclusions:**

Birth by unplanned, but not planned, caesarean section was associated with poorer maternal adjustment and instrumental birth was associated with lower maternal satisfaction with labour and birth. These relationships were found to be partially mediated by psychosocial variables. Psychosocial interventions in the perinatal period should be considered to optimise maternal postnatal adjustment.

**Electronic supplementary material:**

The online version of this article (10.1186/s12905-019-0738-x) contains supplementary material, which is available to authorized users.

## Background

An increase in births by Caesarean Section (CS) globally, has highlighted concerns that women who experience CS may have poorer physical and psychosocial adjustment after birth [1–3]*.* Physical outcomes have been relatively well documented, however, the relationship between mode of birth and psychosocial outcomes would appear to be complex with studies to date demonstrating mixed findings [4, 5].

A review by Lobel and DeLuca (2007) found that women who deliver by CS had more negative perceptions of their birth experience, themselves and their infants. They demonstrated poorer parenting behaviour and may be at higher risk of emotional distress in comparison to women who had a vaginal birth. These findings have been borne out in further studies with mode of birth being shown to be associated with postnatal depression [6–8], post-traumatic stress disorder [9–11] and poorer adjustment to parenthood [12, 13]. The findings, however, are not consistent and other studies have shown that planned CS has a favourable impact [14] or they have not found an association between mode of birth and these psychosocial outcomes [15–20].

Historically, many of these studies have been small and based on convenience samples and inconsistent findings may reflect these methodological limitations [4]. However, there have been variations in the aspects of mode of birth and how maternal postnatal adjustment has been defined that may explain inconsistencies that continue to be observed. For example, there has been variability in the different categories of mode of birth studied with some studies comparing CS or instrumental birth to spontaneous vaginal birth (SVD) and others focusing on planned versus unplanned birth. Sadat et al. (2014) found no association between mode of delivery and postpartum depression, however CS was not differentiated into planned and unplanned CS [21]. If there is time to plan the procedure for the health of the mother or baby the benefits and risks of a caesarean compared with a vaginal birth will be discussed with a health professional in advance of labour and birth. Unplanned CS occurs when a problem arises such as fetal or maternal distress. As the decision-making and the context of care are very different between planned and unplanned CS it is important to differentiate between them. In an early study, Boyce and Todd (1992) reported that women having an emergency CS had more than six times the risk of developing postnatal depression 3 months postpartum in comparison to women who had vaginal deliveries [22]. Blomquist et al. (2011) found that unplanned CS was associated with dissatisfaction and distress in comparison to planned CS [14]. Rowlands and Redshaw (2012) found that unplanned CS and forceps-assisted vaginal births were associated with poorer health and wellbeing for women, with those experiencing the latter being more likely to report ongoing post-traumatic stress disorder (PTSD) type symptoms several months after the birth [23]. Women who had an instrumental birth have also reported the experience as more traumatic than SVD, with fear of childbirth being more common [24, 25]. Large population-based studies that can identify SVD, instrumental birth and CS and discriminate between planned versus unplanned birth are needed to provide a comprehensive evaluation of what aspects of birth are most important.

Maternal postnatal adjustment, as a psychosocial outcome in these studies, has been variously defined as self-rated health [26], satisfaction [27], psychological distress [7, 11, 28] and adjustment to parenthood [28]. These different aspects of maternal postnatal adjustment, while related, reflect different underlying concepts [29]. Studies would benefit from exploring multiple components of maternal postnatal adjustment rather than focusing on one outcome.

Finally, Carquillat, Boulvain and Guitter (2016) recommend that mode of birth not be considered in isolation when exploring its relationship with maternal postnatal adjustment [27]. A number of variables have been identified that could potentially mediate this relationship. DeLuca and Lobel (2014) reported an association between mode of birth and childbirth satisfaction that was mediated by reduced control and unmet expectations. Other studies have highlighted support and the relationship with the caregiver [30], involvement in decision-making [30], experienced control [31, 32] and the first moments with the newborn [32, 33] as important variables that are directly related to birth method and may mediate the relationship with maternal postnatal adjustment.

There is a good theoretical rationale for exploring the role of each of these factors as mediators. Perceived control is thought to influence adjustment to acute stressful events [34] and the degree to which an event is expected is considered to influence how people cope with it [35]. Both perceived control and expectations may be affected if planned mode of birth changes, resulting in poorer postnatal adjustment. Women’s involvement in decision-making around the time of birth provides an important mechanism for increasing the information they receive and their sense of control over decisions that affect their wellbeing [4, 36]. Social support is considered to be a buffer in acutely stressful events [37] and skin-to-skin contact during first moments with the baby after birth is considered to have a positive impact by elevating oxytocin, which antagonizes the flight-fight effect, decreasing maternal anxiety [38] and may also promote early parenting behaviours [39, 40] including breastfeeding [41].

The 2014 National Maternity Survey is a population based study conducted in England that provides detailed information on women’s experience of care during pregnancy, labour and birth and after birth [33]. It has comprehensive questions on mode of birth, experience of support, involvement in decision-making, satisfaction with care, maternal wellbeing, how long until the mother held her baby, when the mother felt her baby belonged to her and infant health at 3 months. This is a unique dataset to evaluate a model that explores the direct relationship between mode of birth and postnatal maternal adjustment at 3 months and indirect relationships through psychosocial variables. We used this dataset to test the following hypotheses:

1. Mode of birth (SVD, instrumental, planned CS, unplanned CS) is associated with postnatal maternal adjustment as measured by three indicators: a composite postnatal wellbeing measure, maternal satisfaction with care during labour and birth, and maternal/infant sense of belonging.
2. Perceived control, maternal expectation, support in labour, how long until the mother held her baby mediate the relationship between mode of birth and the three indicators of postnatal maternal adjustment listed above.

## Methods

### Design

This study is a secondary analysis of a population-based survey in England, UK in 2014. Content of the questionnaire can be found in the initial survey report [33].

### Sample

The survey sample was made up of a random sample of 10,000 women who had their baby in a two-week period at the beginning of January 2014 in England. Women were selected for the survey by the Office for National Statistics (ONS) from birth registrations. Women were excluded if their baby had died or they were aged less than 16.

ONS provided information on each woman’s age group, marital status, an area-based measure of deprivation (the Index of Multiple Deprivation (IMD) in quintiles), country of birth, and whether or not she had responded to the questionnaire, for this random sample of 10,000 women. This enabled responders and non-responders to be compared. This analysis included all primiparous women with singleton babies who provided information about mode of birth (*n* = 2139).

### Data collection

Women in the sample received a questionnaire 3 months after birth, along with an invitation letter, an information leaflet and information sheet in 19 different languages with a Freephone contact number. Women were offered three ways to respond to the questionnaire 1) a postal questionnaire which could be completed and returned to the National Perinatal Epidemiology Unit (NPEU) in a Freepost envelope 2) to complete the questionnaire online, or 3) to complete the survey by telephone, using a Language line interpreter if needed. The questionnaires, each identifiable only by a unique reference number, were returned by post/online/phone to NPEU and logged. The initial mail out took place in April 2014. A reminder letter was sent out 2 weeks later, a further questionnaire was sent out 4 weeks after the initial mail out and again after 8 weeks, if no response had been received.

### Measures

A 26 page questionnaire was completed which took women through their experience of pregnancy, labour and birth, and postnatal care and allowed them to describe the care they had received. The following items and composite variables were taken from the questionnaire.

#### Mode of birth

Women were asked what kind of birth they had and were given the following reply options: “Normal (vaginal) birth”, “Birth using forceps”, “Birth using vacuum cap on the baby’s head (ventouse)” and “A caesarean (through a cut in the abdomen)”. For caesarean births women were asked if the birth was “Planned and carried out before you went into labour”, “Planned, but carried out after you had gone into labour”, or “The result of an unforeseen problem during your labour”. Due to small group size, forceps and ventouse were combined into ‘Instrumental’, and the two categories of planned caesarean were combined. The numbers in each group can be found in Table 1.

**Table 1:** Sample characteristics (all primiparous women with singletons, with known mode of birth, *N* = 2139)

	Mode of birth									
	SVD		Instrumental		Planned CS		Unplanned CS		Total	
	n	%	N	%	N	%	n	%	n	%
Maternal age***										
16–19	59	5.8	11	2.1	4	2.2	12	3.1	86	4.0
20–24	189	18.4	66	12.3	23	12.5	49	12.5	327	15.3
25–29	352	34.3	173	32.2	34	18.5	117	29.9	676	31.6
30–34	296	28.9	194	36.1	74	40.2	126	32.2	690	32.3
35–39	111	10.8	76	14.2	32	17.4	74	18.9	293	13.7
40+	19	1.9	17	3.2	17	9.2	13	3.3	66	3.1
Total (*Missing = 1)*	1027	100.0	537	100.0	184	100.0	391	100.0	2138	100.0
Index of Multiple Deprivation										
1	185	18.0	116	21.6	32	17.4	76	19.4	409	19.1
2	212	20.6	97	18.1	32	17.4	87	22.3	428	20.0
3	220	21.4	132	24.6	47	25.5	81	20.7	480	22.4
4	219	21.3	122	22.7	38	20.7	73	18.7	452	21.1
5 (most deprived)	191	18.6	70	13.0	35	19.0	74	18.9	370	17.3
Total	1027	100.0	537	100.0	184	100.0	391	100.0	2139	100.0
Ethnicity										
White	865	85.6	466	89.1	151	85.8	326	86.0	1808	86.5
Mixed	27	2.7	8	1.5	2	1.1	7	1.8	44	2.1
Asian	95	9.4	40	7.6	13	7.4	33	8.7	181	8.7
Black	20	2.0	7	1.3	9	5.1	11	2.9	47	2.2
Other	4	0.4	2	0.4	1	0.6	2	0.5	9	0.4
Total (*Missing = 50)*	1011	100.0	523	100.0	176	100.0	379	100.0	2089	100.0
Age left full-time education (years)										
< 17	143	14.1	72	13.4	35	19.6	54	13.9	304	14.3
17–18	268	26.4	140	26.1	47	26.3	111	28.5	566	26.7
19+	601	59.2	325	60.5	97	54.2	225	57.7	1248	58.8
Still in education	3	0.3	0	0	0	0	0	0	3	0.1
Total *(Missing = 18)*	1015	100.0	537	100.0	179	100.0	390	100.0	2121	100.0

* *p* < 0.05, ** *p* < 0.01, *** *p* < 0.001

#### Outcome measures

##### Maternal postnatal wellbeing

A composite score for postnatal wellbeing was made up of the following six health and wellbeing questions that significantly correlated with each other (Cronbach’s alpha 0.66). Higher scores reflect better wellbeing and the maximum score was six.

**Table:** 

*Maternal postnatal wellbeing*	
Depression at 3mths	N = 1, Y = 0
Anxiety at 3mths	N = 1, Y = 0
Fatigue at 3mths	N = 1, Y = 0
PTSD-type symptoms at 3mths. Any 2 of sleep problems, difficulties concentrating, flash-backs	N = 1, Y = 0
Edinburgh Postnatal Depression Scale at 3 mths	</=12 = 1, > 12 = 0
Physical wellbeing at 3mths	very/quite well = 1, else = 0

##### Maternal satisfaction with care during labour and birth

This was a single question which asked: ‘Overall, how satisfied or dissatisfied were you with the maternity care you experienced during your labour and birth’ using a 5 point Likert scale with responses ranging from ‘very satisfied’ through to ‘very dissatisfied’.

##### Maternal/infant sense of belonging

This was one item which asked ‘When did you feel your baby really belonged to you? The response levels were: ‘During pregnancy’, ‘immediately after birth’, ‘in the first few days’, ‘in the first few weeks’, ‘only recently’ and ‘not quite yet.’ It was scored 5 for ‘During pregnancy’ to 0 for ‘Not quite yet’.

#### Mediator variables

##### Perceived control during labour and birth

This was a composite measure of seven questions related to decision-making, receiving information, and control (Cronbach’s alpha 0.81). Each item response was coded 0 or 1 and the overall score was a sum of the item scores, with higher scores indicating greater perceived control (maximum score = 7).

**Table:** 

*Scoring Items 1–3*	
1. I was involved enough in decisions (or didn’t want/need to be)	Agree = 1, agree to some extent or disagree = 0
2. Felt no pressure from healthcare professionals to have interventions	
3. Did not feel out of control during birth experience	
*Scoring Items 4 to 7*	
4. Overall, were you given information about choices for care	Yes = 1, to some extent or no = 0
5. Overall, were you able to participate in decision-making about care	
6. Overall, were you given enough information to decide about care	
7. Overall, were you given information at right time to decide about care	

##### Healthcare professional (HCP) support during labour

This was made up of three items scored 0 or 1 (higher scores indicated greater support, maximum score 3) and had a Cronbach’s alpha of 0.82.

**Table:** 

*Scoring item 1*	
1. Always had confidence and trust in staff during labour and birth	Always = 1, sometimes, rarely or never = 0
*Scoring items 2 and 3*	
2. Felt well supported by staff during labour and birth	1.Agree = 1, agree to some extent or disagree = 0
3. Staff communicated well during labour	

##### Expectations

This variable was made up of one item: ‘Overall how do you feel your labour and birth went?’ with ‘worse than you expected’, ‘more or less as you expected’ and ‘better than expected’ as response options, scored from 0 to 2.

##### Holding the baby

This was an indicator of the time elapsed between the baby’s birth and when the mother first held the baby: ‘Soon after your baby was born, were you able to hold your baby?’ If yes, ‘How soon after birth did you hold you baby?’ This was categorised into 0–2 min, 3–15 min, more than 15 min, and women who did not hold their baby soon after birth, and was coded from 0 to 3 where 0 = did not hold baby soon after birth, 3 = held baby within 2 min.

##### Infant health at 3 months

In addition to the above psychosocial mediators, a binary indicator of infant health was also included ‘Does your baby have any health problems now?’ (coded yes = 0; no = 1). This is in recognition that ongoing concerns about infant health after birth can impact on maternal adjustment at 3 months [42].

### Analysis

Mode of birth was the categorical explanatory variable (SVD, instrumental, planned CS, unplanned CS); the mediator variables included Perceived control, Expectations, Holding the baby, Health care professional (HCP) support, and Infant health at 3 months; and Maternal adjustment was the outcome variable. Maternal adjustment was considered in three ways: Maternal postnatal wellbeing at 3 months (Model 1); Maternal satisfaction with care during labour and birth evaluated at 3 months (Model 2); and Maternal/infant sense of belonging (Model 3). Separate analyses were conducted for each measure of maternal adjustment.

**Fig. 1:**
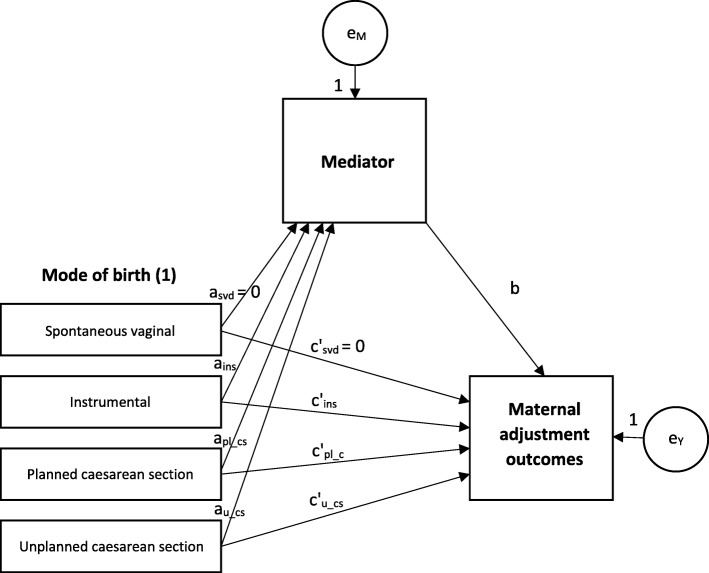
Mediation model for mode of delivery and maternal adjustment outcomes

The mediation analysis followed the Baron and Kenny method [43]. First, linear regression models were used to explore unadjusted univariable associations between mode of birth and each maternal adjustment outcome (*c*; not shown in Fig. 1) and between mode of birth and each potential mediator (*a* in Fig. 1) as an intermediate outcome. Next, regression models for the association between mode of birth and the maternal adjustment outcomes (*c’* in Fig. 1) adjusting for the potential mediators (*b* in Fig. 1) were fitted. Evidence for statistical significance in the univariable regressions was based on t-tests for continuous variables and the ANOVA F-test for categorical variables. Where the unadjusted effects of mode of birth on potential mediators and on maternal adjustment– paths *a* and *c* in Baron and Kenny’s mediation model – and the adjusted effect of potential mediators on maternal adjustment (path *b*) were statistically significant, the proportion of effect mediated was estimated as the relative change in effect between the unadjusted (*c*) and adjusted effect of mode of birth (*c’*) on maternal adjustment (Fig. 1). Mode of birth was a categorical non-ordinal variable, therefore the proportion of effect mediated for each category of mode of birth was estimated *relative* to SVD in a series of pairwise linear regression models (Hayes & Preacher, 2014). The path coefficients for the comparison group were set to zero. Sobel’s test was used to infer whether the amount of mediation observed was statistically significant [44]. For inference, at least one significant relative mediated effect, based on Sobel’s test, was interpreted as evidence of mediation of the effect of mode of birth on maternal adjustment [45]. We used a 0.05 level of significance for the regression models, but given the relatively large number of independent statistical tests conducted, we used a higher threshold of 0.001 for statistical significance in the tests for mediation [46]. All analyses were conducted using STATA 13 SE.

## Results

Completed questionnaires were returned by 4571 women. Once undeliverable questionnaires were excluded from the denominator this gave a usable response rate of 47%. There was significant under-representation of women who were young, living in deprived areas, and those born outside the UK [33]. However, the survey was well-completed with missing data for individual items ranging from 1.0 to 5%.

The mean age of the baby at questionnaire return was 15.4 weeks (SD = 5.0, range 12–52 weeks for whole sample). The characteristics of the 2139 primiparous women with singleton deliveries for whom mode of birth was reported are shown in Table 1. Overall, less than half (48%) of these women had a SVD, a quarter had an instrumental birth with forceps or ventouse, and just over a quarter (27%) had a CS, mostly due to unforeseen problems.

***Hypothesis 1: Mode of birth (SVD, instrumental, planned CS, unplanned CS) is associated with postnatal maternal adjustment as measured by three indicators: a composite postnatal wellbeing measure, maternal satisfaction with care during labour and birth, and maternal/infant sense of belonging.***

Univariable analysis of the relationship between mode of birth and mediators and mode of birth and maternal adjustment outcome variables can be found in Table 2. There was some evidence of an overall difference in Maternal postnatal wellbeing scores across the categories of mode of birth (F-test *p*-value = 0.0481). Instrumental, planned CS and unplanned CS were associated with lower Maternal postnatal wellbeing scores compared to SVD (Table 2). Maternal satisfaction with care during labour and birth scores also differed across the categories of mode of birth (F-test p-value < 0.001): women who delivered through instrumental, planned CS and unplanned CS reported lower Satisfaction with care during labour and birth scores than those delivering by SVD. However, there was no evidence of association between mode of birth and Maternal/infant sense of belonging (F-test p-value = 0.1667). Thus, further analyses are focussed on Maternal postnatal wellbeing and maternal satisfaction with care during labour and birth (models 1 and 2).

**Table 2:** The association of mediator variables and maternal adjustment outcomes with mode of birth

	Mode of birth									
	SVD		Instrumental		Planned CS		Unplanned CS		Total	
	N	%	n	%	n	%	n	%	n	%
Mediators										
Perceived control – mean (95% CI)***	5.00 (4.87, 5.12)		4.38 (4.19, 4.57)		4.58 (4.24, 4.92)		3.86 (3.63, 4.09)		4.60 (4.51, 4.70)	
Expectations (n, % worse than expected)***	156	15.6	273	52.0	37	21.1	247	65.5	713	34.3
Holding baby (n,% within 2 mins of birth)***	658	71.1	247	49.1	16	9.3	39	10.6	960	48.8
HCP support– mean (95% CI)***	2.49 (2.43, 2.55)		2.26 (2.16, 2.36)		2.52 (2.37, 2.66)		2.09 (1.96, 2.21)		2.36 (2.31, 2.41)	
Infant health at 3 mths (n,% with problems)**	124	12.2	81	15.3	38	21.2	60	15.7	303	14.4
Outcomes - mean (95% CI)										
Maternal postnatal wellbeing*	5.60 (5.55, 5.66)		5.51 (5.43, 5.60)		5.52 (5.35, 5.68)		5.44 (5.33, 5.55)		5.54 (5.50, 5.59)	
Maternal satisfaction with labour and birth***	4.54 (4.49, 4.59)		4.28 (4.19. 4.37)		4.60 (4.48, 4.72)		4.16 (4.05, 4.27)		4.41 (4.37, 4.45)	
Mother/Infant belonging	4.84 (4.76, 4.92)		4.77 (4.65, 4.89)		4.69 (4.49, 4.90)		4.69 (4.54, 4.84)		4.78 (4.72, 4.84)	

* *p* < 0.05, ** *p* < 0.01, *** *p* < 0.001

The association between sociodemographic variables and mode of birth or outcomes was explored. There was strong evidence of association between maternal age and mode of birth (*p* < 0.001) with older mothers more likely to deliver surgically and some evidence of association between Index of Multiple Deprivation and Satisfaction with care during labour and birth (*p* = 0.031), with more deprived women reporting lower satisfaction. Also, age of the baby at the time of questionnaire completion was correlated with greater Maternal postnatal wellbeing (*p* = 0.025). However, overall there were only weak association of the sociodemographic variables with *both* mode of birth and maternal adjustment outcome and they were therefore excluded from further analyses.

***Hypothesis 2: Perceived control, maternal expectation, support in labour, how long until the mother held her baby, and infant health at 3 months mediate the relationship between mode of birth and postnatal maternal adjustment as measured by a composite wellbeing measure and maternal satisfaction with care during labour and birth.***

**Fig. 2:**
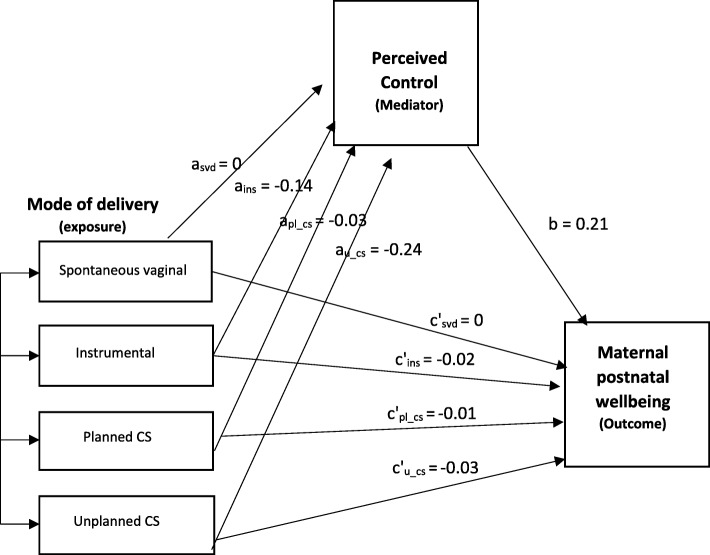
The association between mode of delivery and maternal postnatal wellbeing mediated

The effect of each of the potential mediators on Maternal postnatal wellbeing (Table 3) and Satisfaction with labour and birth (Table 4) is shown separately. Perceived control had the highest mediation effect (76% of total effect mediated, *p* < 0.001) and the mediation model can be found in Fig. 2 (full data for all mediation models can be found in the Additional file 1). Control also mediated between both instrumental and unplanned CS and Satisfaction with care during labour and birth (57 and 69% of total effect mediated, *p* < 0.001 and *p* < 0.001 respectively). There was evidence that Expectations regarding labour and birth mediated the associations between unplanned CS and Maternal postnatal wellbeing (12% effect mediated, *p* = 0.004), and between both instrumental and unplanned CS and Satisfaction with care during labour and birth (50 and 46% effects mediated, *p* < 0.001 and p < 0.001 respectively)***.*** Holding the baby soon after birth significantly mediated the associations between unplanned CS and Maternal postnatal wellbeing (38% effect mediated, *p* < 0.001), and between both instrumental and unplanned CS and Satisfaction with care during labour and birth (8 and 19% effect mediated, *p* < 0.001 and *p* < 0.001 respectively). Similarly, HCP support significantly mediated the association between unplanned CS and Maternal postnatal wellbeing (19% effect mediated, *p* < 0.001), and between both instrumental and unplanned CS and Satisfaction with care during labour and birth (31 and 36% effect mediated, *p* < 0.001 and *p* < 0.001 respectively)***.*** Infant health (ie ‘Does your baby have any health problems now?’ yes or no) was NOT a significant mediator of the association between method of birth and Maternal postnatal wellbeing or Satisfaction with care during labour and birth.

**Table 3:** Unadjusted and adjusted association between mode of birth and Maternal postnatal wellbeing by mediator variable

	Coefficients	Mode of birth (exposure)			
		SVD	Instrumental	Planned CS	Unplanned CS
Maternal postnatal wellbeing	Unadjusted (c)*	0	− 0. 09 (− 0.19 to 0.02)	− 0.09 (− 0.25 to 0.07)	− 0.16 (− 0.27 to − 0.04)
	Standardised	0	− 0.04 (− 0.09 to 0.01)	− 0.01 (− 0.04 to 0.01)	−0.08 (− 0.20 to 0.05)
Mediator					
Perceived control	Adjusted (c’)**	0	–	–	− 0.07 (− 0.19 to 0.05)
	Standardised	0			−0.03 (− 0.08 to 0.03)
	% Effect mediated		–	–	76.0
	Sobel p-value		–	–	< 0.001
Expectations	Adjusted (c’)**	0	–	–	− 0.08 (− 0.20 to 0.05)
	Standardised	0			−0.02 (− 0.09 to 0.02)
	% Effect mediated		–	–	11.6
	Sobel p-value		–	–	0.004
Holding the baby	Adjusted (c’)**	0	–	–	−0.04 (−0.18 to 0.10)
	Standardised	0			−0.02 (− 0.08 to 0.05)
	% Effect mediated		–	–	38.1
	Sobel p-value		–	–	< 0.001
HCP support	Adjusted (c’)**	0	–	–	−0.11 (−0.23 to 0.00)
	Standardised	0			−0.05 (− 0.10 to 0.00)
	% Effect mediated		–	–	18.7
	Sobel p-value		–	–	< 0.001
Infant health at 3 mths	Adjusted (c’)**	0	–	–	−0.16 (−0.27 to − 0.04)
	Standardised	0			−0.07 (− 0.13 to − 0.02)
	% Effect mediated		–	–	1.8
	Sobel p-value		–	–	0.106

*p* values * < 0.05, ** < 0.001

**Table 4:** Unadjusted and adjusted association between mode of birth and Satisfaction with care during labour and birth by mediator variable

	Co-efficient	Mode of birth (exposure)			
		SVD	Instrumental	Planned CS	Unplanned CS
Satisfaction with care during labour and birth	Unadjusted (c)*	0	− 0.26 (− 0.36 to − 0.16)	0.06 (− 0.09 to 0.21)	−0.38 (− 0.49 to − 0.27)
	Standardised	0	− 0.13 (− 0.17 to − 0.18)	0.01 (− 0.01 to 0.03)	−0.18 (− 0.23 to 0.13)
Mediator					
Perceived control	Adjusted (c’)**	0	−0.15 (− 0.23 to − 0.06)	–	− 0.13 (− 0.23 to − 0.03)
	Standardised	0	−0.07 (− 0.12 to − 0.03)		−0.07 (− 0.12,-0.02)
	% Effect mediated		56.6	–	69.4
	Sobel p-value		< 0.001	–	< 0.001
Expectations	Adjusted (c’)**	0	0.02 (−0.08 to 0.12)	–	−0.00 (− 0.12 to 0.11)
	Standardised	0	0.02 (−0.03 to 0.07)		−0.01 (− 0.07 to 0.04)
	% Effect^†^ mediated		49.8	–	46.0
	Sobel p-value		< 0.001	–	< 0.001
Holding the baby	Adjusted (c’)**	0	−0.19 (−0.30 to − 0.09)	–	−0.22 (− 0.35 to − 0.08)
	Standardised	0	− 0.10 (− 0.15 to − 0.05)		−0.10 (− 0.17 to − 0.04)
	% Effect^†^ mediated		8.4	–	19.4
	Sobel *p*-value		< 0.001	–	< 0.001
HCP support	Adjusted (c’)**	0	−0.11 (−0.18 to − 0.04)	–	−0.11 (− 0.19 to − 0.03)
	Standardised	0	− 0.05 (− 0.08 to 0.02)		−0.06 (− 0.10 to − 0.02)
	% Effect^†^ mediated		30.7	–	36.0
	Sobel p-value		< 0.001	–	< 0.001
Infant health at 3 mths	Adjusted (c’)**	0	−0.24 (−0.34 to − 0.14)		− 0.37 (− 0.48 to − 0.26)
	Standardised	0	−0.12 (− 0.17 to − 0.07)		−0.17 (− 0.22 to − 0.12)
	% Effect mediated		0.5	–	0.4
	Sobel p-value		0.127	–	0.125

*p* values * < 0.05, ** < 0.001

## Discussion

Hypothesis 1 was partially supported in that there was a difference between modes of birth and maternal postnatal adjustment as measured by Maternal postnatal wellbeing and Satisfaction with care during labour and birth. Women who had an unplanned CS had the poorest postnatal adjustment. Mode of birth was not associated with Maternal/infant sense of belonging.

Hypothesis 2 was also partially supported as four out of the five proposed mediation variables showed partial mediation of the relationship between mode of birth and both Maternal postnatal wellbeing and Satisfaction with care during labour and birth. The strongest mediator was Perceived control and the only variable not to show a significant mediation effect was Health of the infant at 3 months.

These findings highlight the importance of investigating psychosocial variables and how they relate to physical birth outcomes, such as mode of birth, as well as how psychosocial variables help to explain the impact of birth experience on subsequent outcomes, especially maternal postnatal adjustment. The findings are largely in keeping with previous studies that have looked at planned versus unplanned CS birth and in particular a recent study [47] with 3006 first time mothers that found that women who had unplanned caesarean birth had the least positive feelings overall in comparison to those who delivered by SVD, instrumental birth or planned CS. These women were also more likely to feel disappointed and a failure.

There is a significant literature that demonstrates experienced control is highly correlated with birth experience [29, 31, 48–53]. This study goes a step further to demonstrate how mode of birth, mediated by Perceived control, is associated with Maternal postnatal wellbeing and Satisfaction with care during labour and birth. Qualitative research suggests that unplanned CS and instrumental birth have been associated with feelings of helplessness and loss of control [32] and women who have experienced instrumental birth also report feeling traumatised [25, 54]. Women who had experienced an instrumental birth have described how co-operation with hospital staff and understanding what was occurring were key factors in feeling involved and in control [54]. Our findings suggest that identifying ways to help women maintain or regain a sense of control during birth or after birth is a key component of care to support maternal postnatal adjustment.

In this study, maternal expectations were also found to mediate the relationship between mode of birth and Maternal postnatal adjustment. Some studies recommend that better preparation of women and their partners through antenatal education may be beneficial in helping women have realistic expectations about birth [32]. However maternal expectation has not always been found to predict birth experience [31, 55]. In addition, antenatal education was found to have no effect on women’s experiences of birth [56] and a negative association between attending antenatal classes and birth experience has also been reported [57]. Therefore, further consideration needs to be given to the content, timing and relevance of antenatal education in regard to mode of birth.

Carquillat, Boulvain and Guittier found that women who delivered by CS experienced more negative first moments with their newborn in comparison to women who delivered vaginally [27]. Guittier et al. 2014 reported that women who had a CS felt deprived of the sensory discovery of their baby immediately after birth [32]. DeLuca and Lobel (2014) found that the most powerful predictor of satisfaction with childbirth was how soon after birth the mother held her baby [29]. Our findings confirm that holding the baby is an important and simple action that supports both satisfaction with care during labour and birth and maternal postnatal wellbeing. While it is not always possible for the woman to hold her baby straight after birth, these findings suggest women should be supported in having as much skin-to-skin contact as is feasible. It also should be noted that there is limited evidence of the impact of skin-to-skin contact at birth on the longer term maternal-child relationship [58] and preparing parents beforehand may help them think of ways of compensating for any immediate sense of loss of this experience [32] and promoting their relationship with their baby.

Finally, HCPs have an important role to play in the experience of women who have an unplanned birth. The very nature of unplanned birth produces a lack of continuity and challenges effective communication. Having a HCP whose primary role is to support a woman and her partner through this process may be an important mechanism for improving postnatal adjustment [50, 51, 55].

### Strengths and limitations

The main strength of the study is the use of a large and diverse population-based sample. We were able to account for CS being planned or unplanned and to look at different aspects of maternal adjustment including maternal postnatal wellbeing, satisfaction with care during labour and birth and maternal/infant sense of belonging. By conducting a mediation analysis we were able to explore the relationship between psychosocial factors, mode of birth and maternal postnatal adjustment, highlighting the complexity of these relationships and illustrating the need for providing both physical and psychological interventions at this time to optimise maternal and infant postnatal health and wellbeing.

There were a number of potential methodological limitations. The response rate was 47% and women who were younger, living in deprived areas and born outside the UK were significantly less likely to respond. We do not have psychological wellbeing data for non-responders so cannot comment if women with mental health problems were less likely to respond. The survey was conducted at 3 months postpartum with data about labour and birth being reported retrospectively. While variables such as mode of birth are likely to be accurately reported, more subjective variables such as Satisfaction with care during labour and birth may be biased by conditions at the time of reporting. However, completing the survey at 3 months postpartum avoids the initial emotions that can characterize the early postpartum period (the ‘halo’ effect), and minimises socially desirable responses by completing the questionnaire independently from the care provider [53, 59]. The Hayes and Preacher (2014) approach facilitated the analysis of mode of birth as a categorical non-ordinal variable but did not permit analysis of multiple mediators in the same model [45]. A further limitation is that some of the mediators and outcomes were not validated, standardised measures and some relied on single items. It is possible that the lack of association between mode of birth and maternal-infant relationship may be related to how this was measured.

### Implications for research and practice

Women who have an unplanned birth, particularly unplanned CS, may need additional support to promote postnatal wellbeing and maternal satisfaction with labour and birth. There are many ways that women can be supported, for example, providing information around birth that helps parents with their expectations. Increasing support during labour and birth [60], informing and engaging with women and their partners [55] and establishing skin-to-skin contact as soon as possible after birth may all help to promote postnatal wellbeing and satisfaction [1]. It would be possible to introduce interventions at a number of different time points to optimise care for women who experience unplanned birth. However, it is currently unclear what would be most effective in improving postnatal adjustment and proposed interventions should be investigated using robust prospective methods.

## Conclusions

Birth by unplanned CS is associated with poorer maternal adjustment as measured by a composite Maternal postnatal wellbeing variable. Unplanned caesarean section and instrumental birth are associated with lower maternal satisfaction with labour and birth at 3 months postpartum. The relationship was found to be partially mediated by a number of psychosocial variables. Consideration needs to be given to how to enhance support for women throughout pregnancy, labour and birth and the postpartum to promote a sense of control, a trusting relationship with their HCPs and adequately informed expectations around the time of birth. Psychosocial support interventions to promote maternal adjustment following unplanned birth need to be evaluated to identify an optimal approach to care.

## Additional file

Additional file 1: **Table S1.** a: Mediation by **Perceived control** of the association between mode of delivery and maternal adjustment. b: Mediation by **Expectations** of the association between mode of delivery and maternal adjustment. c: Mediation by **Holding baby** of the association between mode of delivery and maternal adjustment. d: Mediation by **HCP support** of the association between mode of delivery and maternal adjustment. (DOCX 28 kb)

## Abbreviations

CS: Caesarean Section
HCP: Health care professional
IMD: Index of Multiple Deprivation
PTSD: Post Traumatic Stress Disorder
SVD: Spontaneous Vaginal Delivery
