# Supplementary material for: Psychosocial factors that mediate the association between mode of birth and maternal postnatal adjustment: findings from a population-based survey
Source: BMC Womens Health. 2019 Mar 4;19:42. doi: 10.1186/s12905-019-0738-x (PMC6399915; doi:10.1186/s12905-019-0738-x)
Supplement: Supplementary file 1 — Table S1. a: Mediation by Perceived control of the association between mode of delivery and maternal adjustment. b: Mediation by Expectations of the association between mode of delivery and maternal adjustment. c: Mediation by Holding baby of the association between mode of delivery and maternal adjustment. d: Mediation by HCP support of the association between mode of delivery and maternal adjustment. (DOCX 28 kb) [file 12905_2019_738_MOESM1_ESM.docx]

**Additional file 1**

Table S1a: Mediation by **Perceived control** of the association between mode of delivery and maternal adjustment

| **Outcomes** | | **Mode of delivery (exposure)** | | | | **Control (mediator) (b)** |  |
| --- | --- | --- | --- | --- | --- | --- | --- |
|  |  | Spontaneous vaginal | Instrumental | Planned CS | Unplanned CS |  |  |
| **Mediator** | | | | | | | |
| **Control** | Unadjusted *β* (a)** | 0 | -0.61  (-0.83, -0.39) | -0.41  (-0.76, -0.06) | -1.12  (-1.38, -0.87) |  |  |
|  | Unadjusted, standardised *β* (a)** | 0 | -0.14  (-0.18, -0.09) | -0.03  (-0.05, -0.01) | -0.24  (-0.29, -0.18) |  |  |
| **Maternal adjustment outcome** | | | | | | | |
| Maternal postnatal wellbeing | Unadjusted *β* (c)* | 0 | -0. 09  (-0.19, 0.01) | -0.09  (-0.25, 0.07) | -0.16  (-0.27, -0.04) |  |  |
|  | Unadjusted, standardised *β* (c)* | 0 | -0.04  (-0.09, 0.01) | -0.01  (-0.04, 0.01) | -0.07  (-0.13, -0.02) |  |  |
|  | Adjusted *β* (c’)** | 0 | -0.04  (-0.13, 0.06) | -0.04  (-0.19, 0.12) | -0.07  -0.19, 0.05 | 0.09  (0.07, 0.11) |  |
|  | Adjusted, standardised *β* (c’)** | 0 | -0.02  (-0.07, 0.03) | -0.01  (-0.03, 0.02) | -0.03  (-0.09, 0.03) | 0.21  (0.17, 0.26) |  |
|  | % Effect mediated |  | - | - | 76.0 |  |  |
|  | Sobel p-value |  | - | - | <0.001 |  |  |
|  |  |  |  |  |  |  |  |
| Satisfaction with care during labour and birth (5 cats) | Unadjusted *β* (c)** | 0 | -0.26  (-0.36, -0.16) | 0.06  (-0.09, 0.21) | -0.38  (-0.49, -0.27) |  |  |
|  | Unadjusted, standardised *β* (c)** | 0 | -0.13  (-0.18, -0.08) | 0.01  (-0.01, 0.03) | -0.08  (-0.23, -0.13) |  |  |
|  | Adjusted *β* (c’)** | 0 | -0.15  (-0.23, -0.06) | 0.16  (0.04, 0.29) | -0.13  (-0.23, -0.03) | 0.21  (0.19, 0.23) |  |
|  | Adjusted, standardised *β* (c’)** | 0 | -0.07  (-0.12, -0.03) | 0.03  (0.01, 0.05) | -0.07  (-0.12, -0.03) | 0.48  (0.45, 0.52) |  |
|  | % Effect^†^ mediated |  | 56.6 | - | 69.4 |  |  |
|  | Sobel p-value |  | <0.001 | - | <0.001 |  |  |

F-test p values

* <0.05 ** <0.001 †Mediation *relative* to comparison group

‘-‘ indicates that adjusted *β *not calculated as unadjusted *β* was not significant

Table S1b: Mediation by **Expectations** of the association between mode of delivery and maternal adjustment

| **Outcomes** | | **Mode of delivery (exposure)** | | | | **Expectations (mediator) (b)** |
| --- | --- | --- | --- | --- | --- | --- |
|  |  | Spontaneous vaginal | Instrumental | Planned CS | Unplanned CS |  |
| **Mediator** |  |  |  |  |  |  |
| **Expectations** | Unadjusted *β* (a)** | 0 | -0.68  (-0.76, -0.60) | -0.06  (-0.18, 0.06) | -0.93  (-1.02, 0.85) |  |
|  | Unadjusted, standardised *β* (a)** | 0 | -0.38  (-0.42, -0.33) | -0.01  (-0.03, 0.01) | -0.49  (-0.53, -0.44) |  |
| **Maternal adjustment Outcome** | |  |  |  |  |  |
| Maternal postnatal wellbeing | Unadjusted *β* (c)* | 0 | -0.09  (-0.19, 0.02) | -0.09  (-0.25, 0.07) | -0.16  (-0.27, -0.04) |  |
|  | Unadjusted, standardised *β* (c)* | 0 | -0.04  (-0.09, 0.01) | -0.01  (-0.04, 0.01) | -0.07  (-0.13, -0.02) |  |
|  | Adjusted *β* (c’)** | 0 | -0.03  (-0.14, 0.08) | -0.10  (-0.25, 0.06) | -0.08  (-0.20, 0.05) | 0.08  (0.03, 0.14) |
|  | Adjusted, standardised *β* (c’)** | 0 | -0.02  (-0.07, 0.04) | -0.02  (-0.04, 0.01) | -0.03  (-0.09, 0.04) | 0.09  (0.04, 0.13) |
|  | % Effect mediated |  | - | - | 11.6 |  |
|  | Sobel p-value |  | - | - | 0.004 |  |
|  |  |  |  |  |  |  |
| Satisfaction with care during labour and birth (5 cats) | Unadjusted *β* (c)** | 0 | -0.26  (-0.36, -0.16) | 0.06  (-0.09, 0.21) | -0.38  (-0.49, -0.27) |  |
|  | Unadjusted, standardised *β* (c)** | 0 | -0.13  (-0.18, -0.08) | 0.01  (-0.01, 0.03) | -0.08  (-0.23, -0.13) |  |
|  | Adjusted *β* (c’)** | 0 | 0.02  (-0.08, 0.12) | 0.10  (-0.03, 0.22) | -0.00  (-0.12, 0.11) | 0.41  (0.36, 0.46) |
|  | Adjusted, standardised *β* (c’)** | 0 | 0.01  (-0.03, 0.06) | 0.02  (-0.00, 0.04) | -0.02  (-0.08, 0.04) | 0.37  (0.33, 0.41) |
|  | % Effect^†^ mediated |  | 49.8 | - | 46.0 |  |
|  | Sobel p-value |  | <0.001 | - | <0.001 |  |

F-test p values

* <0.05 ** <0.001 † Mediation *relative* to comparison group

‘-‘ indicates that adjusted *β *not calculated as unadjusted *β* was not significant

Table S1c: Mediation by **Holding baby** of the association between mode of delivery and maternal adjustment

| **Outcomes** | | **Mode of delivery (exposure)** | | | | **Holding baby (mediator) (b)** |
| --- | --- | --- | --- | --- | --- | --- |
|  |  | Spontaneous vaginal | Instrumental | Planned CS | Unplanned CS |  |
| **Mediator** |  |  |  |  |  |  |
| **Holding baby** | Unadjusted *β* (a)* | 0 | -0.32  (-0.40, -0.24) | -1.11  (-1.23, -1.00) | -1.13  (-1.21, -1.04) |  |
|  | Unadjusted, standardised *β* (a)* | 0 | -0.18  (-0.22, -0.13) | -0.20  (-0.22, -0.18) | -0.59  (-0.63, -0.55) |  |
| **Maternal adjustment outcome** | |  |  |  |  |  |
| Maternal postnatal wellbeing | Unadjusted *β* (c)* | 0 | -0.09  (-0.19, 0.02) | -0.09  (-0.25, 0.07) | -0.16  (-0.27, -0.04) |  |
|  | Unadjusted, standardised *β* (c)* | 0 | -0.04  (-0.09, 0.01) | -0.01  (-0.04, 0.01) | -0.07  (-0.13, -0.02) |  |
|  | Adjusted *β* (c’)** | 0 | -0.05  (-0.15, 0.06) | 0.01  (-0.17, 0.19) | -0.04  (-0.18, 0.10) | 0.11  (0.05, 0.18) |
|  | Adjusted, standardised *β* (c’)** | 0 | -0.02  (-0.07, 0.03) | 0.00  (-0.03, 0.03) | -0.02  (-0.09, 0.05) | 0.11  (0.06, 0.15) |
|  | % Effect mediated |  | - | - | 38.1 |  |
|  | Sobel p-value |  | - | - | <0.001 |  |
|  |  |  |  |  |  |  |
| Satisfaction with care during labour and birth (5 cats) | Unadjusted *β* (c)** | 0 | -0.26  (-0.36, -0.16) | 0.06  (-0.09, 0.21) | -0.38  (-0.49, -0.27) |  |
|  | Unadjusted, standardised *β* (c)** | 0 | -0.13  (-0.18, -0.08) | 0.01  (-0.01, 0.03) | -0.08  (-0.23, -0.13) |  |
|  | Adjusted *β* (c’)** | 0 | -0.19  (-0.30, -0.09) | 0.21  (0.05, 0.36) | -0.22  (-0.35, -0.08) | 0.14  (0.09, 0.20) |
|  | Adjusted, standardised *β* (c’)** | 0 | -0.10  (-0.15, -0.05) | 0.03  (0.01, 0.06) | -0.11  (-0.17, -0.04) | 0.14  (0.10, 0.19) |
|  | % Effect^†^ mediated |  | 8.4 | - | 19.4 |  |
|  | Sobel p-value |  | <0.001 | - | <0.001 |  |

F-test p values

* <0.05 ** <0.001 † Mediation *relative* to comparison group

‘-‘ indicates that adjusted *β *not calculated as unadjusted *β* was not significant

Table S1d: Mediation by **HCP support** of the association between mode of delivery and maternal adjustment

| **Outcomes** | | **Mode of delivery (exposure)** | | | | **HCP support (mediator) (b)** |
| --- | --- | --- | --- | --- | --- | --- |
|  |  | Spontaneous vaginal | Instrumental | Planned CS | Unplanned CS |  |
| **Mediator** |  |  |  |  |  |  |
| **HCP support** | Unadjusted *β* (a)** | 0 | -0.22  (-0.33, -0.11) | 0.04  (-0.14, 0.21) | -0.39  (-0.51, -0.26) |  |
|  | Unadjusted, standardised *β* (a)* | 0 | -0.10  (-0.15, -0.05) | 0.00  (-0.02, 0.02) | -0.17  (-0.22, -0.12) |  |
| **Maternal adjustment outcome** | |  |  |  |  |  |
| Maternal postnatal wellbeing | Unadjusted *β* (c)* | 0 | -0.09  (-0.19, 0.02) | -0.09  (-0.25, 0.07) | -0.16  (-0.27, -0.04) |  |
|  | Unadjusted, standardised *β* (c)* | 0 | -0.04  (-0.09, 0.01) | -0.01  (-0.04, 0.01) | -0.07  (-0.13, -0.02) |  |
|  | Adjusted *β* (c’)** | 0 | -0.07  (-0.17, 0.03) | -0.10  (-0.25, 0.06) | -0.11  (-0.23, 0.00) | 0.13  (0.09, 0.17) |
|  | Adjusted, standardised *β* (c’)** | 0 | -0.03  (-0.08, 0.02) | -0.02  (-0.04, 0.01) | -0.05  (-0.11, 0.00) | 0.15  (0.11, 0.20) |
|  | % Effect mediated |  | - | - | 18.7 |  |
|  | Sobel p-value |  | - | - | <0.001 |  |
|  |  |  |  |  |  |  |
| Satisfaction with care during labour and birth (5 cats) | Unadjusted *β* (c)** | 0 | -0.26  (-0.36, -0.16) | 0.06  (-0.09, 0.21) | -0.38  (-0.49, -0.27) |  |
|  | Unadjusted, standardised *β* (c)** | 0 | -0.13  (-0.18, -0.08) | 0.01  (-0.01, 0.03) | -0.08  (-0.23, -0.13) |  |
|  | Adjusted *β* (c’)** | 0 | -0.11  (-0.18, -0.04) | 0.07  (-0.03, 0.17) | -0.11  (-0.19, -0.03) | 0.65  (0.62, 0.68) |
|  | Adjusted, standardised *β* (c’)** | 0 | -0.05  (-0.08, -0.02) | 0.01  (-0.00, 0.03) | -0.06  (-0.10, -0.02) | -0.17  (-0.22, -0.12) |
|  | % Effect^†^ mediated |  | 30.7 | - | 36.0 |  |
|  | Sobel p-value |  | <0.001 | - | <0.001 |  |

F-test p values

* <0.05 ** <0.001 †Mediation *relative* to comparison group

‘-‘ indicates that adjusted *β *not calculated as unadjusted *β* was not significant
